# Sexual life and fertility desire in long-term HIV serodiscordant couples in Addis Ababa, Ethiopia: a grounded theory study

**DOI:** 10.1186/1471-2458-12-900

**Published:** 2012-10-24

**Authors:** Tewodros G Hailemariam, Getnet M Kassie, Mitike M Sisay

**Affiliations:** 1 Department of Public Health, Faculty of Health Sciences, Wolaita Sodo University, Wolaita Sodo, Ethiopia; 2 School of Public Health, Addis Ababa University, Addis Ababa, Ethiopia

## Abstract

**Background:**

Even though remarkable progress has been achieved, HIV/AIDS continues to be a major global health priority. HIV discordant relationship is one of the emerging issues in HIV prevention endeavour. In Ethiopia, very little is known about HIV-serodiscordant couples particularly how they manage their sexual relationship and fertility desire. Therefore, we conduct this study with the aim of exploring the experiences of HIV discordant couples about their sexual life, and fertility desire in the context of long-term relationships in Addis Ababa, Ethiopia.

**Methods:**

A grounded theory approach was employed using in-depth interviews among 36 informants in ART/PMTCT centers of three public hospitals, a health center and one PLHIV association in Addis Ababa. Theoretical sampling was used to recruit 28 clients who lived in a discordant relationship and eight health care providers as key informants. Data collection and analysis were undertaken simultaneously using a constant comparison. The analysis was facilitated using OpenCode software.

**Results:**

A grounded theory pertaining to sexual life and desire to have a child among HIV discordant couples emerged as “maintaining the relationship” as a core category. Couples pass through a social process of struggle to maintain their relationship. The causal conditions for couples to enter into the process of struggle to maintain their relationship were collectively categorized as “Entering in-to a transition” (knowing HIV serostatus) and this includes mismatch of desire to have a child, controversy on safe sex versus desire to have a child, and undeniable change in sexual desire and practice through time were the features in entering into-transition. Then after the transition, couples engaged in certain actions/strategies that are categorized as “dealing with discordancy” such as entertaining partner’s interest by scarifying once self interest to maintain their relationship.

**Conclusions:**

HIV discordant couples’ relationship is filled with controversies of maintaining relationship versus fear of getting infected. The findings of this study have suggested the need to view discordant couple’s actions as a process of maintaining their relationship in the context of eminent risks. Further study should be done among HIV discordant couples to assess the fitness of the current model in different setups and population. In addition, a study could begin to test the hypotheses proposed in this study.

## Background

HIV and AIDS continue to be a major global health priority despite the promising progress that has been made so far in preventing the disease. In our planet, an estimated 33 million people are living with human immunodeficiency virus (HIV) and 2.7 million new infections occur each year [1,2]. Unfortunately, sub-Saharan Africa shares a lager burden of this pandemic where the main driving force in transmission of HIV remains heterosexual intercourse [2,3].

However, heterosexual couples who live in HIV-serodiscordants status may not engage in recommended sexual practice that can protect the HIV negative partner because of various reasons. For instance, studies from USA and Nigeria showed that discordant couples didn’t use condom during sexual intercourse due to their desire to maintain primary relationship, establish trust and increase intimacy [4,5]. Moreover, some findings indicate that the desire to have a child among PLHIV is increasing as a result of access to antiretroviral therapy (ART) and prevention of mother-to-child transmission (PMTCT) [6].

In Ethiopia, though the estimated adult HIV prevalence is lower compared to other sub-Saharan African countries, but it is still one of the countries with largest HIV infected population [7]. Despite the complexity of the dynamics and the huge burden of the HIV epidemic in Ethiopia, the challenge related to HIV-serodiscordant couples in relation to its role in the transmission of HIV infection versus desire for children and sexual life is not well explored. Data are limited with regards to the magnitude and roles of serodiscordant couples. A study done in Bahir Dar Town (in Amhara Region-Ethiopia) has shown that the prevalence of HIV-serodiscordant status among couples attending VCT was 9.8% [8].

These all indicates the relevance of looking for more information to understand how serodiscordant couples manage their relationships in the face of knowing their HIV status. It is important for policy makers, researchers, program implementers, and practitioners to know how discordant couples actually manage their sexual and fertility desire. Therefore, we have conducted this study with the aim of exploring the experiences couples’ on sexual life, and the desire to have child/children in the context of long-term HIV discordant relationships.

## Methods

### Qualitative research approach

In this study, we have used a qualitative research method employing the Grounded Theory. Grounded theory is a research tool which enables to find out and conceptualise the latent social patterns and structures in a study of interest through the process of constant comparison. It is a way of thinking and studying social phenomena and as the same time it provides techniques and procedures for gathering and analysing data that aims to generate a theory explicitly from data collected using methods such as observation, document analysis and interviews, with the theory built through inductive reasoning [9-12].

### Settings

We conducted the study in clinics providing ART/PMTCT services at three selected public hospitals (Zewditu Memorial, St. Paul, and Yekatit 12 Hospitals), one health center and also one PLHIV association in Addis Ababa from December 2010 to April 2011.

Addis Ababa is the capital city of Ethiopia and the seat of African Union. The city has a population of around three million, which is administratively divided into 10 sub-cities and 116 Woredas/ districts. According to 2007 single point HIV prevalence estimate the prevalence of HIV in the city was 9.2% for 2010 [13].

### Study participants

In this study we recruited a total of 36 informants from two groups; individuals who live in HIV-serodiscordant relationship (Table 1) and key informants such as health professionals who particularly involve in the process of counselling and care giving for HIV-serodiscordant couples at ART or PMTCT unites including nurses, medical doctors and health officers (Table 2).

**Table 1:** Background information of research participants from discordant relationship, December 2010 - March 2011 (n=28)

**Characteristics of participants**		**Frequency**
Sex	Male	15
	Female	13
Age	35 years old and below	13
	Above 35 years old	15
Duration since being married/in Union	5 Years and below	5
	Above 5 years	23
Educational level	Illiterate	1
	Primary education (1-8grade)	8
	Secondary education [9-12]	12
	Tertiary and above (college & above)	7
Employment Status	Employed at formal sector	18
	Self employed	3
	Unemployed	7
HIV serostatus	HIV positive	19
	HIV negative	9
Duration since knowing HIV test result	1 – 5 years	12
	Above 5 years	16

**Table 2:** Key informants involved in in-depth interview from different health setup in Addis Ababa, December 2010 - March 2011 (n=8)

** ID**	**Sex**	**Age**	**Profession**	**Level of Education**	**Position**	**Experience (Year)**
HP1	Female	45	Medical Doctor	MD	Head, CDC Unit	7
HP2	Male	29	Nurse	BSc	ART specialist	3
HP3	Female	36	Nurse	Diploma	Drug adherence and Follow-up Counselor	4
HP4	Female	24	Health Officer	BSc	Practitioner at ART center	2
HP5	Male	48	Medical Doctor	MD	Practitioner at ART Center	5
HP6	Male	34	Nurse	BSc	ART specialist at ART	3
HP7	Female	25	Health Officer	BSc	Practitioner at ART Center	3
HP8	Female	29	Patient Expert (Peer educator)	Diploma	Drug adherence and Follow-up Counselor	3

HIV-serodiscordant couple participants were eligible for the study, when they were 18 years (cohabiting or marriage below the age of 18 is not legally supported) or older and self-reported being in HIV-serodiscordant relationship, at least one year in the sexual union/relationship after finding out his/her HIV status, and who consented to participate in the study.

Key informants (health care providers) were also enrolled in the study based on their experiences and involvement in treating people living with HIV and their willingness to participate in the study. Initially we interviewed key informants to have an in-depth understanding of various concepts related to discordant couples.

In selecting the study participants, the principal investigator (PI) approached participants who were identified by health care providers at each setting as having long-term HIV discordant relationships. In some cases, both couples were identified by health care providers when they both visit the facilities. On the other hand, where HIV negative partners didn’t visit the health facilities with their positive spouses an invitation letter with a consent form was sent to the spouse. This was done after having discussion and oral consent with HIV positive partner visiting at the facilities.

The selection process was simultaneous which is characterized by analyzing the data after the first data are gathered and then emerging concepts/categories were included which in turn leads to more data collection to understand the concepts/categories generated deeply. This process of data collection continued until it reached the point of saturation [9].

### Data collection and analysis

The interviews were prescheduled and took place in rooms at the clinics and in the PLHIV association’s offices that guarantee optimum privacy. On average it lasted about 1hr and 15min to carry out an in-depth interview.

Initially we interviewed health care providers composed of different health professionals. Based on the concepts elaborated further on our first interviews we continued the interview with individuals who live in a discordant relationship.

Data collection and analysis were undertaken simultaneously in line with the looping nature of qualitative research method. All interviews which were audio taped and field note of the interviews were fully transcribed to Amharic (the Ethiopian official language) then translated into the English language.

The PI transcribed and translated all the recorded interviews. On average an hour long interview took about six hours to transcribe and five hour to translate. Before the analysis repeated reading of the transcribed data to immerse and familiarize ourselves with the data was done. Finally, the data were analyzed using grounded theory constant comparison approach based on Strauss and Corbin’s recommendation [9].

The process of analysis proceeds with open coding, identifying concepts, categories, properties/subcategories, and emergent storyline integrated using axial coding model. First, the PI read the complete transcripts and generates a list of codes. Then after aggregating and defining concept PI develop memos which can elaborate the concepts/categories developed. Finally integration of categories were done which is linking categories around a core category and refining and trimming the resulting theoretical construction using techniques of rereading memos and raw data (immersed in the data), creating a story line/descriptive sentences, doing diagrams and plain thinking. To manage the overall coding process ‘Opencode software’ was used [9].

### Ethical issues

The research and ethical committee of the School of Public Health at Addis Ababa University approved the study and ethical clearance was obtained from Institutional Review Board of St. Paul Hospital and Addis Ababa Regional Health Bureau as it was required by respective institutions. Moreover, verbal informed consent was obtained from all informants who participated in the study after explaining the purpose of the study in the local language. Participation in the study was voluntary and privacy of individuals and confidentiality of the information was assured both during and after data collection. All of the participants were informed about their right to resign from being part of the study.

### Trustworthiness

To maintain the trustworthiness of the study, we tried to follow rigorous criteria, using several strategies. To see the credibility of the study, we invited some participants to review the findings and ideas which they think correctly represents their point of views were taken for the study. Moreover, the judgment of the transferability of the theory to a new set of situations depends on the contextual information provided by the investigator, thus in this report we hope there is a rich description that can help reader to understand the circumstances.

The idea of dependability includes the consistency with which the data have been analyzed and the theory developed [14,15]. Theoretical sampling was used so that we can collect the data from different angle that will triangulate the information during the analysis with information generated from health professionals. Moreover, debriefing sessions with public health postgraduate students from different experiences were used to check the consistency between the analyzed data and the theory developed.

The confirmability of the findings are an easily met criteria in trustworthiness of findings since the criteria that can be used to make sure the confirmability of finding in grounded theory was not taken as necessary one. The aim of doing grounded theory is not to justify, prove or affirm anything rather develop a living theory that can explain the phenomena well and able to be modified when it is needed [11]. Thus in this research we did an explorative study and explained the core phenomena well. Moreover, at the end we left open the stage on the proposed theoretical explanation for further modification of the model.

## Results

In this study one core category and five sub-categories emerged with some properties. The core category was ‘*struggle to maintain relationship*’ and the five conceptual categories were ‘*entering in to transition*’, ‘*dealing with discordancy*’, ‘*shared life*’, ‘*couple’s Cosmo*’ and ‘*ups and downs*’. Each category is presented in detail with appropriate descriptions and quotes cited in the text to support the categories using elements of paradigm model which is analytical tool [9-11].

### Maintaining the relationship – core category 1

This is the central phenomenon or category which is defined as the process by which couples in discordant relationship strive to sustain their union/marriage. After couples know their HIV status as discordant, they tried different strategies to sustain their relationship and overcome some challenges that threaten their relationship. In this research, the goal that couples had in mind to their actions and strategies towards maintaining their relationship was directed to strengthen family integrity and avoid disruption particularly among couples who had children. One of our key informants tried to express this concept as follows:

*“. . . I know a couple when I was working at PMTCT unit; they stay together for about five years. The wife [who is HIV positive] gave birth to her first child in such circumstances and now she is also pregnant with good health condition. What I’m able to observe from this couple is that the husband [who is sero-negative] is stronger than her. He supports her nicely and he said “where can I go without her?” (H7-*Health officer*)*

**Figure 1:**
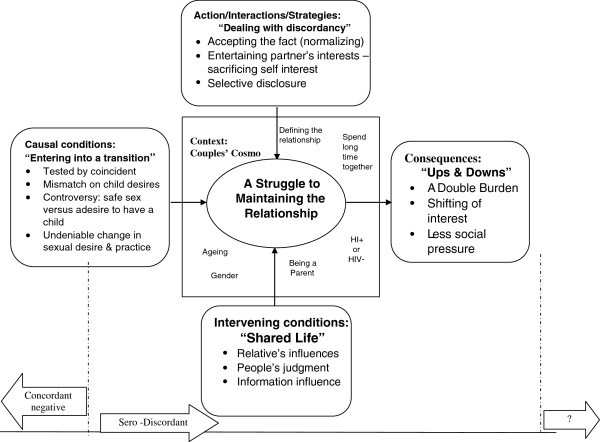
Theoretical model representing the process of maintaining Relationship in the context of long-term HIV discordant relationship.

Maintaining the relationship is carried out mainly through some important actions/interactions or strategies. Thus, couples in such circumstance pass through a process of struggle to maintain their relationship as depicted in Figure 1.

### Entering into a transition - causal conditions

In this study, the causal conditions are defined as the events that occur in the life course of couple’s relationship which came before the decision “Maintaining the Relationship” [9,10]. It is a phase that couples shifted from their previous life experience to the new one due to their HIV test result. It includes some sub-categories/properties.

#### Tested by coincident

Knowing their HIV serostatus is a point where all the events in couple’s life start. As per informants from this study, most couples visit health facilities to be tested for HIV by some coincidence such as ill-health condition, pregnancy or visa processing to travel abroad. These circumstances commonly enforce couples to be tested for HIV which usually involves individual testing. One of our informants whose husband is HIV positive indicated the situation as follows:

“In the first place we were not tested together for HIV…. I had a chance to go abroad and was tested about three or four times. But, for the first time I knew my HIV negative status when I was pregnant. As you know, when you plan to give birth, you are expected to give blood sample for HIV test . . .” (A 33years old HIV negative woman)

#### Mismatch in desire on having a child

After couples enter into “the transition”, one important issue that couples are experiencing is “mismatch on desire to have a child”. Our key informants were indicating that “HIV sero-status” become one important issue on their desire to have children. This condition put couples in a position of struggle to maintain their relationship. A 29 years old HIV positive informant who was a peer educator at health centre indicated the situation as follows:

*“. . . Currently we are using condoms. But he [my husband] has an interest to add one more child . . . because he is not still infected with the virus he makes things difficult for me . . .”* (HP 8-Peer educator)

#### Controversy: safe sex versus a desire to have a child

The other causative condition that couples found themselves in the middle of struggle is controversy between a desire to have a child and having a safe sex practice. Even though, this is expected among HIV negative individuals, it was also a challenge among HIV positive individuals too. For instance, one of our next HIV negative informant told us her experience, since she has a strong desire to conceive a child she was engaged in unprotected sex which later brought a concern of infection. She said;

“You know, I love if I could be able to have another child and breast feed him. However, we stopped having sex for a long time [about four or five years]. We just decided to give more attention to our child. He may not feel anything and me too, but some times when I think about it, I pray to God by saying ‘please don’t let me down, let me have sex without condoms so that I might be able to get a baby’, but I failed. Then I stopped.” (A 33years old HIV negative woman)

#### Undeniable change in sexual desire and practice

Change in sexual desire and practice of individuals in their relationship was another sub-category which emanates from this study. Couples’ sexual desire and practices were not the same when compared with before and after knowing their HIV serostatus. A 29 years old HIV positive woman described the change as follows:

“Previously I used to have very strong desire for sex, but now after we know that my husband and my son are free of the virus, I am not happy when we do it [the sex].” (A 29 years old HIV+ woman)

### Dealing with discordancy - action/interactions/strategies

It refers responses made by individuals or groups to situations, problems, happenings and events [9]. Categories pertaining to activities couples do or events that couples attempt to use to sustain their partnership in the process of struggle to maintain the relationship. Data coded into this category support couples’ intended or manipulated events or actions.

#### Accepting the fact - normalizing

A particular strategy that comes before all other actions taken by individuals who live in a discordant relationship was “accepting the fact”. This strategy starts at the beginning of the transition. As per our key informants indicated, mostly when couples come together to be tested for HIV, those who are HIV negative will take an immediate action of is “leaving his/her partner” there at VCT centers. But, the discordant couples we interviewed indicated that due to some conditions couples were not separated or they want to maintain their relationship after convincing themselves. Thus, this strategy marks differences between those who decide to separate and those who still live together in a discordant relationship. For instance one of our key informants explained the situation as follows:

“….most of the time, when discordant couples make a decision to stay together after they know their serostatus, it is with full knowledge and belief that they would be able to handle whatever challenge that will come in their lifetime. . .” (HP6 - Nurse, ART specialist)

In fact there are some conditions that determined couples’ decision to accept their test result and to stay together in their relationship despite all the pressure that surrounds them. These conditions are discussed below under context or intervening conditions.

#### Entertaining partner’s interests – sacrificing self interest

Even though HIV negative partners may feel they are losing their freedom, the value they have regarding relationship/marriage in general helped them to maintain their relationship. But, they characterize this as “a sacrifice” to the value they hold as described by one of our informants whose experience is presented below:

“ hmm …. In order to keep her [his wife] propensity, willingly I am scarifying to the extent of putting myself on fire. I stopped using condoms because I have observed some negative emotional expressions on her; instability and the like thus we spend five years without using condoms, but God protected me till now.” (A 37 years old HIV negative man)

In addition to unprotected sexual practice, individuals are also engaged in other actions /interactions/strategies under the concept of “entertaining partner’s interest”. For instance, one of our participants also gave us some description on how much deeply he wants to “entertain his partner’s interest” as much possible. Since his spouse has a strong desire to have a child in spite of his concern infection. If she insists to get what she wants he is ready for it.

“. . . … I don’t know, may be in the future we might have the treatment or if the time comes that gives us a chance to have a baby, or if there are different ways; even it could be by paying some money or others I will do it. In addition, if it doesn’t affect her and the baby that will be born, and also if the alternative is only through unsafe sex, I told her that I’m willing” (A 45years old HIV+ man)

According to this man, “maintaining the relationship” is more important than passing the infection and additional financial costs. Couples may have different interests and desires like that of the other group of the society, but here they try to give a priority for their union than other desires. Thus, individuals living in such circumstances are sacrificing their self interest which is an interest related with either sexual, fertility, or career to maintain their relationship.

#### Selective disclosure

Another strategy that is used by couples is “selective disclosure”. All of the research participants agreed on one thing about disclosing HIV test result to others which is to be selective. In general, they don’t disclose their serostatus to anyone due to stigma. However, if they decide to disclose their status, then they will be very selective. One of our key informants, a medical doctor, describes the condition like this:

“. . . these couples' secret is kept by themselves and no one knows about it . . . and this helps them to reduce the psycho-social pressure . . .well in the other corner there are some couples who disclose to some individuals like children, brothers and sisters, but it doesn’t go beyond . . ." (HP1-A Medical doctor)

### “Shared life: living with the community”- intervening conditions

In the paradigm model, these conditions provide the broader structural context in which the actions/interactions/strategies to manage the central phenomenon occurred [9,10]. These are the factors that emerge in relation to couple’s social structure. In this research, these factors are aspects pertaining to close relatives, community, information and their influences.

#### Influences related to family members and close relatives

It is defined as a context in which couple’s relatives or close families interference or pressure on their decision regarding certain matters. This context has a potential to impact couple’s actions and emotions pertaining to the central phenomena. One of our key informants tries to state the influence of relatives on discordant couples as follows:

“The other thing is relatives, you know, they have a chance of hearing about what has happened. Particularly relatives of spouses who are HIV negative would prefer the dissolution of such relationship. If the partner who is negative (he/she) has children from pervious relationship, then, these children would prefer the termination of the marriage. This is just to protect their parent from the infection (may be their father or mother). . .” (HP4 - Health Officer)

Since the couples have shared their life with their relatives to some extent, the pressure that comes from such structure can change their desire towards certain matters within their relationship. Such socio-structural context explains why one person has a certain outcome or chooses certain set of strategies while another person not.

#### People’s judgment – a concern

It is defined as how people view being HIV positive as well as being a discordant couple. The perspective that a community has towards HIV positive individuals and discordant relationships create a different context in a discordant relationship. This pushes couples to engage or prefer certain actions or come up with certain outcomes in their relationship. For instance, one of HIV positive women felt that people’s attitude towards them is not positive thus she chose to keep her status a secret. And she explained it as follows:

“ . . . Since we are hearing things; and this disease is transmitted via sexual intercourse, that’s why I don’t want people to know about my status. If I did sleep with other men, I could not hide my status, because it had resulted from what I did.” (A 35years old HIV+ Woman)

Moreover, fear of people’s judgment was also observed among informants who were HIV negative too. This is because of the fact that they are living with their HIV positive spouse and also if the positive status of their partner is disclosed, then, everybody may assume that they are concordant couples. This was explained by one of our informant as follows:

“. . . I do not know, maybe they see him on the media when he teaches, but I’m not sure about that. Actually I don’t like this thing [his teaching]. Sometimes he exposes himself on media, may the Lord protect me from that. .. I do not like what he is doing. Because, I have kids; for me I do not have any problem, but for our children oh!!!, there may be some discrimination. If people say to these kids that your parents are like this and that then they might be hurt psychologically. Therefore, at least until my children reach adulthood, I don’t want anyone to know about it . . .” (A 27years old HIV negative woman)

#### Information influences

The other intervening condition that explains why individuals or couples as a group have a certain set of strategies in the process of maintaining their relationship is information obtained mainly from counselling services at health facilities. One of our key informants summarized the use of information by victims of HIV as follows:

". . . Well, with regard to sexual activity, they have less sexual interaction or engagement. I think this could be due to some factors mainly related to repeated counselling and advices about HIV such as the transmission, risk and so on. . . Therefore, I think this has influenced their sexual engagement." (HP5 – Medical doctor)

Therefore, if either of them or both of them were able to be exposed to such information, then it would have an intervening role in the couple’s decisions with regard to their sexual life and their desire to have a child.

### “Our Cosmo”: Couple’s living circumstances – context

Context represents structural conditions that shape the nature of situations, circumstances, or problems to which individuals respond by means of action/interaction/emotions [9]. In our research those structural conditions are undertaken in the management of central phenomenon. These are immediate structural conditions to their relationship circumstance which shapes the central phenomenon–maintaining the relationship through selecting a certain strategy or end up with certain consequences.

There are different properties (sub-categories) identified under this category. For instance: “the way couples define their relationship”, and knowing “being in a different HIV serostatus than their partner”. Some HIV positive individuals were considering themselves as “being favoured” by their partner whereas others take it as a very challenging environment due to of the difference in serostatus. This was described by a 29 years old HIV positive woman:

"For me, I don’t prefer being in discordant relationship. If you are not in similar status then it is very difficult. To speak honestly, it is not good because if we had similar serostatus then we could support each other. For instance, if I forgot taking my medicine then he would reminds me and even he wouldn’t ask me to conceive a baby since he would knew the problem. Therefore, from my experience I prefer for others to be with a person that has similar HIV serostatus . . ." (HP8-peer educator)

In addition to this “spending longer time” in the current relationship and “ageing” were other properties under this category that shapes the nature of the situation. Time was observed as a big issue in making certain decision by these couples depending on how long they stay together or how old they were. As a result, they compromise their choices or decisions. This was described by one of our informants as follow:

““. . .at that time [the time of testing] we were more concerned about our future & our children because I’m approaching 50 and my wife was around 45. . . . You see, spending 32 years together is not simple thing. We also have children. As I told you, our mind is not busy on sexual desires, but we do have an option of using condoms if we want.” (A 50years old HIV+ Man)

Moreover, “being parents” and “gender” also play their role in shaping the nature of situation, or circumstances. The desire to have children in case of couples who already have children was relatively low compared to those couples who don’t have one. Having children is another main reason for some couples to maintain their relationship despite other challenges they had. One of our research participants described below how having a child within the relationship shape her decisions.

“. . . You see – my daughter loves her father very much - more than anything. She has something special for him. Therefore, she is the only reason that enforced me to stay with him. It is not his behaviour that makes me to stay.” (A 33years old HIV negative woman)

Even though individuals from discordant couples didn’t say directly anything about gender issue, but the influence of man on female to change her decisions or affecting her negotiation ability on certain issues in additions to other cofactors which could be economical dependency were observed throughout the interaction we have with the informants. This fact were strengthen by one our key informants:

“. . . Speaking about their psychological condition, it is very difficult, since they have somehow tough life. Most of the time, especially females are paying a huge share of the burden especially if they are HIV positive . . . But this is not true for all discordant couples because there are some couples regardless of gender and sero-status, they live together accepting the situation. . .” (HP3)

### ‘Ups and Downs’ - consequences

This category also consists of sub-categories/properties that explain the outcomes of couples’ actions and strategies.

#### A double burden

It is a psychological pressure which is resulted due to the presence of the virus with respect to couples strategies used in the process of maintaining their relationship. According to our informants those HIV positive individuals had some concern while they live in a discordant relationship. It is linked with being an index case within the relationship and at the same time the concern towards their own health status as an HIV positive individual.

For instance, an HIV positive informant describes the concept of “being HIV positive” and “having a discordant relationship” as a double burden:

“. . . hmm regarding being happy or not, may be my wife might not be happy because the virus lives with me, within my blood, I am the one accountable for the case. Thus, I might not feel anything [for sex], but my wife’s feelings can be affected . . .” (A 50 years old HIV+ Man)

People who are HIV negative also share such burden from different directions. The psychological burden in case of HIV negative individuals came from the concern they have with regard to seroconversion in the long run and fear of losing their partner. Thus in both cases they are facing a sort of double psychological burden as a result of their actions and context.

#### Shifting of interest

It is an event that occurred throughout the course of relationship after couples knew their HIV serostatus. It is a response to the situation or being in discordant relationship. After the strategy of “accepting the fact” or the decision made to stay together as a discordant couple, considering other conditions, there is a shift of interest after knowing their serostatus. The following informant describes shift of interest as follows;

“. . . After we know [our HIV status], it would not be possible [striving for sex]; it is not the same because she doesn’t have any interest to look for another person except me. Therefore, we made our mind to give more attention to our children; to give them good care, better education and the like. We stopped looking for other things [like sex] and it will not be possible.” (A 41years old HIV+ Man)

Thus the presence of child/children shapes the dimension of the shift of couple’s interest towards their child/children.

#### ‘Less social pressure’

Less social pressure also is another event that couples experience in their course of relationship. It is linked with the action or strategy preferred to use by individuals regarding disclosure status to other people. As a result of their preferences to the “selective disclosure” mentioned earlier, there is no as such undesirable social pressure which could affect their relationship.

Most of our informants disclose their HIV test result to specific close relatives or they don’t disclose it at all. Thus due to their strategy used in the process of maintaining their relationship they didn’t face a significant social pressure that resulted from being HIV positive or being in a discordant relationship. This was explained by one of the respondents as follows:

“..About neighbours, they may guess otherwise no one knows about it. Therefore, we don’t have any problem. We live a smooth social life together, my children spend some time with them; as a result we don’t have any problem. There is no such pressure, they feed and wash my children. We live like the previous time” (A 27 years old HIV negative Woman)

Selective disclosure throughout the process of maintaining relationship resulted with less social pressure on couples or individuals who live in a discordant relationship. Especially if they have children such strategy were taken as important in order to avoid unnecessary pressure which might be imposed on children due to their parents’ HIV serostatus.

## Discussion

In this study “maintaining the relationship” was the emergent core category grounded in the data. This was couples’ main concern in the context of long-term HIV discordant relationship. “Struggling to maintain the relationship” was the basic social process used to deal with HIV discordant relationship which is a forward movement through integrating with its own contexts and conditions. Some finding indicates that in addition to other factors HIV/AIDS bring changes in couple’s life/marriage [16] Another study that has been done in Brazil also indicated that the HIV diagnosis had changed participants sexual trend, according to the women’s responses: they were afraid of infecting their partners, they had many new sources of stress which had made them lose their sexual appetite, or they felt less sensual within themselves [17].

This study showed that couples were not tested with a primary intention of knowing their HIV sero-status. Most of them were tested alone by certain coincidence due to some enforcing conditions like sickness, pregnancy and a plan to go abroad. As a result, the overall struggling process to sustain the relationship that couples established start after they enter into the “transition” i.e., knowing that they have HIV discordant serostatus.

A study conducted in Addis Ababa indicated, couples were not able to be tested for HIV together due to unavailability and/or unwillingness of partners [18]. Moreover, couples voluntary counselling and testing also didn’t get attention in HIV prevention activities in sub-Saharan African countries including Ethiopia [19]. Thus, entering accidentally into a different dimension of relationship becomes a condition that can bring some changes in couple’s perspectives and life since they were not ready for it. The change as observed in different studies as well as in our study, indicate shifts in the desire to have a child and sexual practices [17,20,21].

While couples are passing through the process of the central phenomenon which is maintaining the relationship using selected strategies, these were determined by other intervening conditions such as relatives interferences, people’s judgment due to the perspective they have towards HIV, PLHIV and discordant couples, and information influence. Some authors reported that HIV/AIDS brings changes in a couple’s life [16] and specifically, a study done in Brazil indicated that the HIV diagnosis had changed participants sexual trend and appetite due to concern of infecting their partners. [17].

We have seen the role of relatives on couples desire on having children comparing with the finding from Uganda that indicates pressure from relatives to reproduce were one of the reasons why the couples’ desire to have children in such relationship [20]. Moreover, another cross-sectional study finding from the same country also support this fact that relatives influence was the major factor for couple’s desire to have a child [22]. Our study limited in producing data in relation to the amount of relative’s pressure on serodiscordant couples to have a child. This may be attributed to the selective disclosure preferred by most or as a result of the existing stigma.

Despite the intervening conditions, couple’s living circumstances were playing a significant role in affecting their decisions and certain outcomes in the process of maintaining their relationship. Our study suggested that context of couple’s living circumstances such as the way couples define their relationship, spending longer time together, being an index case or HIV negative, being a parent, ageing and gender play their own roles in shaping couples actions/interactions/strategies in the process of maintaining their relationship. These properties of couple’s context were interlinked with one another.

A study from Northern Thailand showed that time spent in partnership has been identified as one of the context which is valued by couples to maintain their relationship as discordant couples [23,24]. Therefore, this is one of couples’ contexts which affect their decision towards certain actions. Moreover, being an index case or being HIV positive or negative, being parent especially for having children in common, age and gender were another context of couple’s life that shape their desire and preferences in a certain way to some extent.

Therefore, individuals who take into consideration all the conditions and context in regard to their relationship will engage with certain kinds of actions/interactions/strategies that have significant role to play in maintaining their relationship. Some studies from different countries showed similar evidence on couples’ decisions towards a particular strategy or experiencing certain outcomes in their relationships [20,22,23,25-27].

Our findings further indicate that couples use “accepting the fact” as a strategy which is taking HIV test result as normal phenomena. A study done on vulnerability in Brazil among discordant couples showed that there is a naturalization of HIV/AIDS infection among the studied individuals. According to this finding couples were engaged in risky behaviour due to their belief towards the diseases [28].

Another strategy used among couples to maintain their relationships was avoiding disclosing their status completely or selective disclosure to closer family members. This was related to a belief that people around them will stigmatize them or induce other forms of social pressure. Couples in discordant serostatus in our study also believed that this strategy has helped them to maintain their relationship. This finding was supported by other studies where people who disclosed their status faced stigma. Individuals have their own specific criteria for deciding to whom to disclose which were based on their calculation of risks and benefits of disclosure [29-32].

In this study spouses were seen to put their health status at risk to comfort their partners by performing unprotected sex with their HIV positive partners or bearing a child. This was with an intention of sustaining their relationship. This is supported by the study from Uganda which found out that because of fear of losing partners, spouses were engaged in certain actions like unprotected sex and conception to secure their relationship [20].

Through this struggle process to maintain their relationship, couples face some outcomes as a result of their actions/interactions/strategies. One of the outcomes that emerged as a category was “a double burden” that both of the partners were facing with different extent of pressure. This was expressed by the fact that HIV positive individuals were facing different kinds of psychological pressure as a result of their serostatus. Having an already established relationship with an HIV negative partner introduced a huge fear towards transmission of the virus to the uninfected partner on the one hand and a concern about dissolution of the marriage especially among those who had common children.

Other studies also indicated that people with HIV suffer grossly form mixed feelings towards maintaining their relationship and protecting the uninfected partner from the infection. The change in sexual trend and fertility, were indicative of the fact that HIV positive people are facing psychological pressure to deal with the conditions existing around them [16,17,23,25-27].

On the other hand, HIV negative partners also have their own concerns that impose a psychological burden. One thing that differ their pressure was their serostatus. They don’t have a psychological burden in terms of the virus having a concern of survival like that of HIV positive one. But, they also experience some kinds of pressure that is related with fear of being infected compounded with losing partner or dissolution of a family in general.

In addition to the double burden, shifting of interest was observed among couples which is resulted from the overall process of maintaining their relationship. According to the findings, couples’ interest or desires somehow changed or shifted from before entering into such transition to new relationship experiences. Interest like having a child and sexual desires were somehow changed with taking into account their intervening conditions and context.

Various studies indicated the shift of interest that was observed on individuals who live in a discordant relationship in terms of change in their practice like that of sexual and child desire. Thus couples prioritize their interest more for maintaining their relationship rather than being concerned much for other practices (19–21, 44, 45, 54).

Moreover, selective disclosure of their HIV status to other people throughout the process of maintaining the relationship resulted with less social pressure on couples or individuals who live in a discordant relationship, especially if they have children such strategy were taken as important in order to avoid the unnecessary pressure that might be imposed on children due to their parents’ HIV serostatus.

## Conclusions

Our study gives an in-depth understanding of various concepts. However the study is not without limitation. The short period of our study deters us from observing othe concepts which would have provided us with more detail understanding of the the process. If the study had stayed longer; the trajectory would have showed us other dimensions that we might have missed. For instance, we left one concept that is untouched such as “seroconversion”. The other limitation is language, since we did the interviews with local language, there could be some chances of losing the original sense of meaning while translating the content into the English language.

However, it became apparent that couples were more concerned with maintaining their relationship where sexuality and the desire to have children were embedded, thus the struggle to maintain their relationship become the process. Knowing HIV serostatus either alone or somehow together without any readiness brings couples to the first steps of the process.

Couple’s context such as defining the relationship, spending long time together, being in different HIV serostatus than partner, being parents, ageing and gender contribute their role in shaping couples actions and strategies preferred to pursued. However, by the way of maintaining their relationship within the capsule of their union/marriage, they were engaged in certain risky behaviour like that of having unprotected sex and getting pregnant in order to entertain their partner’s interest.

In addition, to all the intervening conditions their actions and strategies bring some consequences that both or either of them facing throughout the process of maintaining their relationship. Engaging in unsafe sexual practice in addition to other pressures imposed due to different conditions brings a psychological pressure on both of them whereas among those HIV positive there is an additional burden which is related with their serostatus. Ill-health condition was the main concern that creates an additional burden on those HIV positive individuals. Moreover, selective disclosure seems advantageous to overcome and prevent undesirable social pressure.

## Competing interests

The authors declare that they have no competing interests.

## Authors’ contributions

T.G. was responsible for the conception, study design, implementation, data collection, analysis, interpretation and the preparation of the draft manuscript. G.M and M.M involved in the design, the writing, interpretation and critical revision of the paper for intellectual content. All authors read and approved the final manuscript.

## Pre-publication history

The pre-publication history for this paper can be accessed here:

http://www.biomedcentral.com/1471-2458/12/900/prepub
